# Mouse models of hereditary hemorrhagic telangiectasia: recent advances and future challenges

**DOI:** 10.3389/fgene.2015.00025

**Published:** 2015-02-18

**Authors:** Simon Tual-Chalot, S. Paul Oh, Helen M. Arthur

**Affiliations:** ^1^Institute of Genetic Medicine, Newcastle University, Newcastle, UK; ^2^Department of Physiology and Functional Genomics, University of Florida, Gainesville, FL, USA

**Keywords:** arteriovenous malformation, TGFβ signaling, vascular disease, angiogenesis, vascular development, Bmp/Smad signaling

## Abstract

Hereditary hemorrhagic telangiectasia (HHT) is a genetic disorder characterized by a multi-systemic vascular dysplasia and hemorrhage. The precise factors leading to these vascular malformations are not yet understood and robust animal models of HHT are essential to gain a detailed understanding of the molecular and cellular events that lead to clinical symptoms, as well as to test new therapeutic modalities. Most cases of HHT are caused by mutations in either endoglin (*ENG*) or activin receptor-like kinase 1 (*ACVRL1*, also known as *ALK1*). Both genes are associated with TGFβ/BMP signaling, and loss of function mutations in the co-receptor *ENG* are causal in HHT1, while HHT2 is associated with mutations in the signaling receptor *ACVRL1*. Significant advances in mouse genetics have provided powerful ways to study the function of Eng and Acvrl1 *in vivo*, and to generate mouse models of HHT disease. Mice that are null for either *Acvrl1* or *Eng* genes show embryonic lethality due to major defects in angiogenesis and heart development. However mice that are heterozygous for mutations in either of these genes develop to adulthood with no effect on survival. Although these heterozygous mice exhibit selected vascular phenotypes relevant to the clinical pathology of HHT, the phenotypes are variable and generally quite mild. An alternative approach using conditional knockout mice allows us to study the effects of specific inactivation of either *Eng* or *Acvrl1* at different times in development and in different cell types. These conditional knockout mice provide robust and reproducible models of arteriovenous malformations, and they are currently being used to unravel the causal factors in HHT pathologies. In this review, we will summarize the strengths and limitations of current mouse models of HHT, discuss how knowledge obtained from these studies has already informed clinical care and explore the potential of these models for developing improved treatments for HHT patients in the future.

## INTRODUCTION TO HHT AND IMPORTANCE OF MOUSE MODELS

Hereditary hemorrhagic telangiectasia (HHT) is inherited as an autosomal dominant disease and has a prevalence of approximately one case per 5000–8000 individuals (Bideau et al., 1989; Kjeldsen et al., 1999; Dakeishi et al., 2002). Mutations in endoglin (*ENG*) are responsible for HHT1 (McAllister et al., 1994), while mutations in activin receptor-like kinase (*ACVRL1* also known as *ALK1*) are responsible for HHT2 (Johnson et al., 1996). A rare form of HHT disease in which vascular lesions are combined with juvenile polyposis is due to mutations in *SMAD4* (Gallione et al., 2004). Whole genome sequencing will ultimately map the genetic cause of HHT in patients where mutations have not yet been identified. Taken together, HHT1 and HHT2 account for more than 80% of cases of HHT. Consequently, most of the research in understanding this disease has focused on investigating the roles of *ENG* and *ACVRL1* and mouse models carrying mutations in these genes form the focus of this review.

HHT patients develop mucocutaneous lesions known as telangiectases in the nose, mouth, and gastrointestinal (GI) tract, as well as larger arteriovenous malformations (AVMs) in major organs such as the lung, liver, and brain (Shovlin, 2010). The telangiectases are comprised of fragile vessels that are susceptible to rupture and hemorrhage, which means that HHT patients can suffer recurrent anemia following frequent and severe bleeding episodes. Bleeding telangiectases are also difficult to treat clinically and many HHT patients suffer a lifetime of unpleasant disease symptoms. In addition, large AVMs in major organs can pose severe risks to life.

**FIGURE 1:**
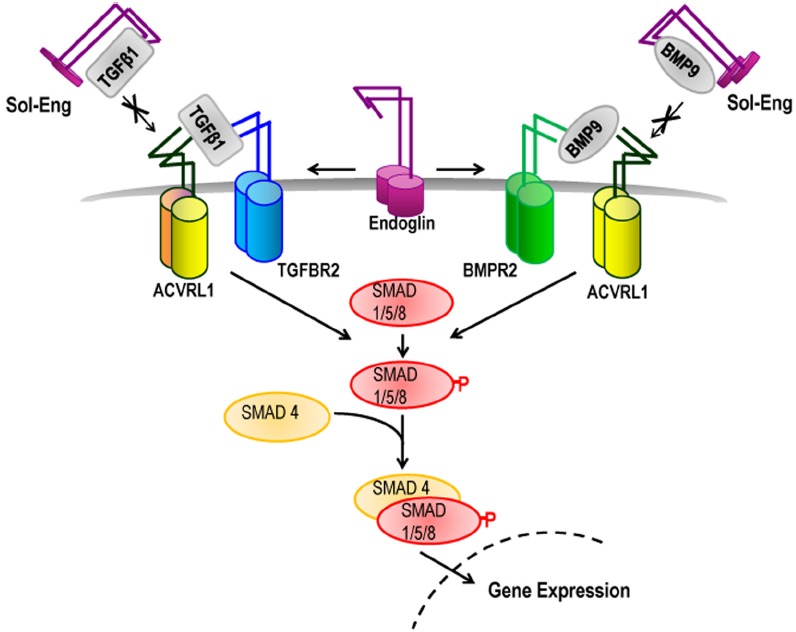
**Role of endoglin and ACVRL1 in TGFβ family signaling.** TGFβ superfamily ligands bind to specific serine/threonine kinase receptors and induce formation of a heteromeric receptor complex. In ECs, TGFβ1 ligand activates ACVRL1/ALK5/TGFBR2 receptor complexes leading to phosphorylation of SMAD1/5/8 which then interacts with SMAD4 protein and moves to the nucleus to regulate transcription of target genes. BMP9 ligand promotes Smad1/5/8-mediated signaling through ACVRL1-BMPR2 receptor complexes. Endoglin promotes signaling through ACVRL1, potentially by facilitating ligand binding to the signaling receptor complex. Under particular environmental conditions (e.g., during inflammation) the extracellular domain of endoglin is cleaved to form soluble endoglin (Sol-Eng), which may sequester circulating BMP9/10 ligands and reduce receptor binding. Sol-Eng has also been shown to inhibit binding of TGFβ to TGFBR2 (Venkatesha et al., 2006) potentially by a sequestering mechanism (as shown).

ENG and ACVRL1 proteins are expressed predominantly in endothelial cells (ECs), but are also found in some other cell types. For example, endoglin is expressed in myofibroblasts, mesenchymal cells, and activated monocytes. Both ENG and ACVRL1 are expressed at the cell surface where they bind selected members of the TGFβ family (Figure 1). ENG is a co-receptor that promotes TGFβ family signaling through the signaling receptor ACVRL1 (Lebrin et al., 2004), and together they can form a protein complex. In this way ENG and ACVRL1 are thought to work together in the same signaling pathway, performing a similar role in the cell, and consistent with the similar clinical phenotype of HHT1 and HHT2. However, there are also some clinical differences between HHT1 and HHT2. HHT1 patients have a higher incidence of pulmonary AVMS and cerebral AVMs, while HHT2 patients are more susceptible to GI bleeding and to liver AVMs (Letteboer et al., 2006; Lesca et al., 2007; Shovlin, 2010; Komiyama et al., 2014). It is not yet clear why these differences occur, but they do point to overlapping, but non-identical functions for ENG and ACVRL1. At present our understanding of disease mechanisms is far from complete and therapies for patients remain limited. Robust animal models of HHT are therefore important (i) to increase our understanding of the disease mechanisms (thereby underpinning the development of new treatments) and (ii) to provide robust models for screening new therapies. In recent years there have been considerable advances in the development of mouse models of HHT and this review summarizes progress to date.

## MICE HOMOZYGOUS FOR NULL MUTATIONS IN *Eng* OR *Acvrl1*

Mice lacking functional endoglin were generated independently by gene targeting in three different laboratories (Bourdeau et al., 1999; Li et al., 1999; Arthur et al., 2000). In all cases, total loss of endoglin expression leads to cardiovascular defects and embryonic death by mid-gestation. Vasculogenesis does not appear to be affected in these endoglin null mice, suggesting that endoglin is not required for initial differentiation of ECs or for formation of a primitive vascular plexus. The yolk sac of *Eng*^–/–^ embryos has enlarged fragile vessels, while the embryo proper shows cardiac cushion defects and delayed maturation of major vessels, with reduced vascular smooth muscle cell (vSMC) coverage. These findings point to the importance of endoglin in angiogenesis and for formation of the primitive cardiac cushions (which are primordia of cardiac valves). Acvrl1 null (*Acvrl1*^–/–^) mice have a very similar phenotype with early embryonic lethality, yolk sac angiogenesis defects, and delayed maturation of the embryonic vessels (Oh et al., 2000). Once again vasculogenesis appears to progress normally suggesting that Acvrl1 is required for angiogenesis, but not for the initial formation of a primitive vascular plexus. In a similar study, embryos lacking *Acvrl1* develop large shunts between arteries and veins, associated with downregulation of genes involved in arterial vessel identity and reduced vSMC coverage (Urness et al., 2000). This led to the initial idea that AVMs in HHT form during development due to fusion of arteries and veins because they have lost their normal molecular identity.

Although the cause of the enlarged fragile vessels typical of *Eng*^–/–^ and *Acvrl1*^–/–^ yolk sacs is unclear, these vessels represent a useful (albeit primitive) model of HHT blood vessels. They were used as a tissue model of HHT1 in an elegant study showing that endoglin is required by ECs to produce locally active TGFβ1 protein (Carvalho et al., 2004). As this EC-derived TGFβ1 protein is critical for differentiation and maturation of the adjacent vSMCs, the reduced TGFβ1 protein defect potentially explains the reason for fragile vessels in HHT. In other words, in the absence of endoglin, there may be insufficient molecular signal (TGFβ1 protein) to permit the normal crosstalk from ECs to the neighboring vSMCs to fully support vSMC maturation and maintain a robust vascular wall (Carvalho et al., 2004). This reduced vessel maturation phenotype is also seen, although much less severely, in the endoglin heterozygous mice (*Eng*^+/–^) and is discussed below.

## HETEROZYGOUS (*Eng*^+/–^ AND *Acvrl1*^+/–^) MOUSE MODELS OF HHT

Heterozygous (*Eng*^+/–^ and *Acvrl1*^+/–^) mice are the closest genetic models of HHT patients in terms of genotype. However, *Eng*^+/–^ and *Acvrl1*^+/–^ mice have a very mild phenotype and HHT-like features appear at low frequency (Bourdeau et al., 1999; Srinivasan et al., 2003; Torsney et al., 2003). Although initially disappointing for those of us working to develop a robust animal model of HHT, this finding is important because it suggests that additional triggers are required for development of HHT and if these triggers can be minimized then HHT patients will have a reduced risk of disease symptoms. The effect of environmental triggers has mainly been tested in *Eng*^+/–^ mice, but we will first discuss the mild baseline defects in the *Eng*^+/–^ and *Acvrl1*^+/–^ mice and then the potential environmental triggers for development of HHT-like lesions.

### BASELINE DEFECTS IN *Acvrl1*^+/–^ AND *Eng*^+/–^ MICE

*Acvrl1*^+/–^ mice have dilated vessels and HHT-like vascular lesions in liver, nailbed, intestine, or skin that develop between 7 and 20 months (Srinivasan et al., 2003). However, these lesions occur at incomplete penetrance (approximately 40% *Acvrl1*^+/–^ mice have one or more lesions) and in an unpredictable manner. Heterozygous *Eng*^+/–^ mice also show a range of HHT-like vascular lesions including nosebleeds, ear telangiectasias, and vessel dilation that develop between 3 months and 2 years. HHT-like lesions are more frequent in the 129/Ola genetic background (affecting approximately 40–50% *Eng*^+/–^ animals) suggesting that genetic modifiers may play a role in susceptibility to HHT disease (Bourdeau et al., 1999; Torsney et al., 2003). Importantly, these mice also show reduced vascular smooth wall coverage of postcapillary venules that is also more severe in the 129/Ola genetic background (Torsney et al., 2003). As reduced vSMC coverage of vessels is expected to increase susceptibility to hemorrhage, a landmark study set out to test whether thalidomide, a vascular maturation factor, would be beneficial for treating HHT (Lebrin et al., 2010). Initially using the *Eng*^+/–^ mouse model, thalidomide treatment was shown to restore the reduced vSMC coverage of vessels. More importantly, HHT patients treated with thalidomide for 3 months also show reduced severity of epistaxis associated with improved vSMC coverage of nasal mucocutaneous vessels (Lebrin et al., 2010). These findings point to the value of using thalidomide in the clinic for treating severe epistaxis in HHT. However, there is little known about the long term benefits of thalidomide treatment in HHT and thalidomide also carries high health risks due to its major teratogenic and irreversible neuropathic effects. Therefore, safer derivatives of thalidomide are needed before this can be taken forward to clinical trials.

### POTENTIAL TRIGGERS OF VASCULAR ABNORMALITIES IN *Eng*^+/–^ MICE: INFLAMMATION AND ANGIOGENESIS

An early study suggested that inflammation may be a trigger for HHT, as bleeding lesions are more pronounced in the eyelids of *Eng*^+/–^ mice with blepharitis (Torsney et al., 2003). Moreover there is evidence suggesting that *Eng*^+/–^ mice are less able to resolve inflammatory stimuli, which would mean a prolongation of this trigger (Jerkic et al., 2010; Shen et al., 2014). This inflammatory environment also has an effect on the local level of endoglin protein. The extracellular domain of endoglin is cleaved from the cell surface by proteolysis (Hawinkels et al., 2010) and the released soluble endoglin protein (Sol-Eng) may act as a “ligand sink” by binding circulating selected TGFβ family ligands to disrupt the normal co-auxiliary receptor function of endothelial endoglin. In fact, increased levels of Sol-Eng are observed in patients with sporadic (non-familial) cerebral AVMs suggesting excess Sol-Eng may disrupt local vessel organization (Chen et al., 2009). Furthermore, endoglin is cleaved from the surface of ECs following stimulation with the inflammatory protein TNFα (Li et al., 2003). Therefore, reduced reservoirs of endoglin protein on ECs (typical of HHT1 patients) combined with inflammatory triggers would increase the risk of generating ECs with insufficient or even no endoglin protein during the inflammatory period. This transient so-called “endoglin protein null” phenotype would increase the risk of vascular lesion development. Therefore anti-inflammatory therapies such as anti-TNFα that would decrease the risk of endoglin protein shedding may be beneficial in treating HHT1. It is not yet clear whether an anti-inflammatory approach would also be useful for HHT2 as further work needs to be done on *Acvrl1*^+/–^ mice to identify potential triggers of vascular abnormalities.

As discussed above, heterozygous (*Eng*^+/–^ and *Acvrl1*^+/–^) mice develop relatively normal blood vessels suggesting there are no major vascular defects during developmental angiogenesis. This also agrees with the fact that the majority of the vasculature in HHT patients functions normally while vascular lesions are localized and sporadic. However, reduced angiogenesis in adult *Eng*^+/–^ mice has been reported in pathological conditions such as femoral artery ligation (Jerkic et al., 2006), myocardial infarction (van Laake et al., 2006), or stroke (Shen et al., 2014). Moreover, in a series of studies focusing on the brain vasculature in *Eng*^+/–^ and *Acvrl1*^+/–^ mice, transient pro-angiogenic stimulation generates abnormal vascular outcomes. In the first study, stimulation of cerebral vasculature with adenoviral particles expressing VEGF in *Eng*^+/–^ mice leads to increased vascular dysplasia but not overt AVMs (Xu et al., 2004). Likewise, intra-cerebral delivery of adeno-associated virus expressing VEGF results in cerebrovascular dysplasia in both *Eng*^+/–^ and *Acvrl1*^+/–^ mice, but with a higher degree of vascular dysplasia and a higher capillary density in *Eng*^+/–^ mice compared with *Acvrl1*^+/–^ mice (Hao et al., 2010). These studies show that angiogenic stimulation is required for the development of vascular dysplasia in heterozygous models. Taken together these studies support the premise that normal levels of endoglin and Acvrl1 are required for typical angiogenic responses and that angiogenesis represents a likely trigger for the formation of abnormal vessels in HHT. However, heterozygosity for *Eng* or *Acvrl1* mutations, even in combination with pro-angiogenic triggers, was not sufficient to trigger AVM formation suggesting that a further event was required.

## *Eng*-INDUCIBLE KNOCKOUT (iKO) AND *Acvrl1*-iKO MICE—MODELS OF HHT1 AND HHT2

**FIGURE 2:**
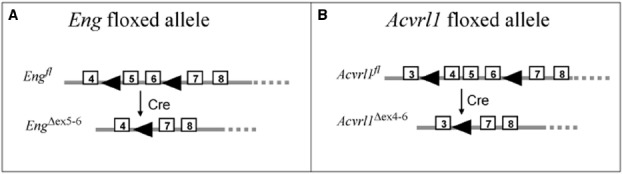
**Floxed mice used to generate models of HHT.** The floxed endoglin mouse **(A)** and the floxed Acvrl1 mouse **(B)** were generated for conditional knockout studies using Cre-LoxP recombination (Allinson et al., 2007; Park et al., 2008). The floxed endoglin mouse **(A)** was designed with the position of loxP sites (arrowheads) to allow conditional deletions of exons (boxed regions) 5 and 6 of the Endoglin gene, which also leads to a frameshift mutation in exon 7 to generate a truncated non-functional protein. Cre mediated recombination of the floxed *Acvrl1* allele **(B)** leads to removal of exons 4–6 which includes the essential transmembrane domain. To achieve cell-type specific knockdown of endoglin or *Acvrl1*, these floxed mice were crossed with specific Cre-lines (see Tables 1 and 2).

To evaluate whether losing the remaining functional *Eng* or *Acvrl1* allele in postnatal life leads to a better mouse model of HHT pathology, genetic tools such as Cre-lox technology have been used to deplete (or “knock out”) the target genes (*Eng* or *Acvrl1*) at a time of choice (Figure 2). In contrast to mice that are heterozygous for mutations in either *Eng* or *Acvrl1*, which overall have only mild vascular defects, homozygous deletions of *Eng* or *Acvrl1* using cell type-specific or time-dependent Cre drivers result in consistent and robust AVMs resembling those seen in HHT patients. Importantly, however, these AVMs are generally limited to sites where there is a pro-angiogenic or inflammatory environment, as described in the following sections.

### PRO-ANGIOGENIC OR INFLAMMATORY STIMULI ARE REQUIRED FOR THE DEVELOPMENT OF AVMs IN *Eng* OR *Acvrl1*-DEFICIENT SKIN AND BRAIN

**FIGURE 3:**
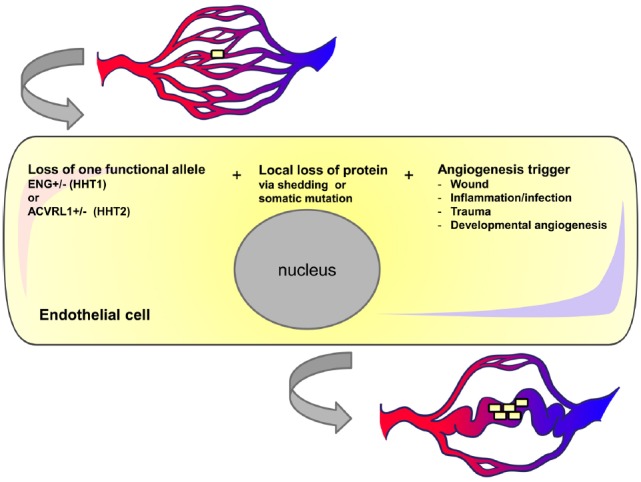
**Three event hypothesis for AVM formation in HHT.** Individual endothelial cells in HHT patients may undergo local loss of endoglin or ACVRL1 protein either due to shedding or somatic mutation. If vessels containing these transient or permanent null cells are exposed to pro-angiogenic triggers then the cells may undergo further proliferation and contribute to vessel enlargement. We suggest that once blood flow increases in this enlarged vessel there is a positive feedback loop leading to further endothelial cell proliferation and vessel enlargement.

Homozygous loss of the *Acvrl1* gene in adult life is required but is not sufficient to trigger AV shunts in cutaneous vessels. An additional environmental insult is required; for example dermal wounding leads to AVMs at the wounded site in *Acvrl1*-deficient mice (Park et al., 2009). These “wound-induced AVMs” also occur in *Eng*-deficient mice (Choi et al., 2014; Garrido-Martin et al., 2014). Furthermore, local application of molecular stimulators of angiogenesis or inflammation such as VEGF or LPS triggers the formation of skin AVMs, while VEGF neutralizing antibody is able to reduce wound-induced AVMs (Han et al., 2014). These results suggest that two genetic hits (loss of both *Eng* alleles, or loss of both *Acvrl1* alleles) combined with environmental pro-angiogenic triggers are necessary for AVM development, the so-called “three event hypothesis” (Figure 3). This three hit paradigm was also observed for brain AVMs in a series of studies delivering local intra-cerebral injection of a Cre expressing adenovirus into the basal ganglia of adult mice carrying floxed *Eng* (*Eng^fl/fl^*) or floxed *Acvrl1* (*Acvrl1^fl/fl^*) alleles. This approach generated regional loss of the target *Eng* or *Acvrl1* gene, which alone had minimal effect on the vasculature. However, when angiogenesis is triggered by local injection of adeno-associated viral vectors expressing VEGF, abnormal vessels with arteriovenous shunting develop that resemble the human brain AVM phenotype (Walker et al., 2011; Choi et al., 2012; Chen et al., 2013). Interestingly, more dysplastic vessels are induced per copy of *Eng* deletion than per copy of *Acvrl1* deletion (Choi et al., 2012), consistent with the observation that HHT1 patients have a higher incidence of brain AVMs than HHT2 patients (Letteboer et al., 2006; Komiyama et al., 2014). All of these mouse studies (Park et al., 2009; Walker et al., 2011; Choi et al., 2012, 2014; Chen et al., 2013; Garrido-Martin et al., 2014; Han et al., 2014) support the idea that a combination of events are needed for AVM formation; first loss of *Acvrl1* or *Eng* protein (either by a second genetic hit or a proteolytic mechanism) followed by a pro-angiogenic or inflammatory trigger; for example, by exposure to VEGF or wounding (Figure 3).

### ENDOTHELIAL-SPECIFIC LOSS OF *Eng* OR *Acvrl1* PREDISPOSES VASCULATURE TO AVM FORMATION

In order to determine which are the critical cell types where Eng and Acvrl1 play their essential roles in vascular development and maintenance, a range of different transgenic Cre lines have been used to conditionally deplete *Eng* or *Acvrl1* in different cell types. Subsequently, vessel remodeling in response to a pro-angiogenic or inflammatory stimulus can be evaluated in each mouse line. Key findings from these *in vivo* studies are summarized in Tables 1 and 2. It is clear that the key cell type is the *endothelial cell*: loss of Acvrl1 or endoglin protein from vascular endothelium is a prerequisite for AVM formation whereas loss of these genes in other cell types (vSMCs, pericytes, or macrophage) has no detectable effect on AVM formation. It is also important to note that the pro-angiogenic signals vary in these different mouse models: in some cases investigations have focused on normal developmental angiogenesis in early postnatal life, while in other cases experiments have used wounding or inflammation as environmental insults to trigger vascular remodeling in adult mice.

**Table 1:** **Summary of HHT phenotypes in conditional endoglin knockout mouse models with different Cre lines**.

**Cre line**	**Targeted cell type (Cre expression)**	**HHT phenotype**	**Reference**
Cdh5-Cre-^ERT2^	ECs (Cre activated in neonate or adult).	*Neonatal*- retinal AVMs *Adult* -enlarged subdermal veins adjacent to matrigel plugs containing VEGF+FGF	Mahmoud et al. (2010)
R26-Cre^ER^	All cell types	AVMs -dependent on VEGF stimulation or wounding	Choi et al. (2014), Garrido-Martin et al. (2014)
SM22α-Cre	vSMCs and ECs	Sporadic AVMs and micro-hemorrhage in brain and spinal cord. 50% mortality by 6 wks	Choi et al. (2014)
LysM-Cre	Macrophage	No phenotype detected	Choi et al. (2014)
Scl-Cre^ER^	ECs	Skin AVMs following wounding	Garrido-Martin et al. (2014)
Myh11-Cre^ER^	vSMCs	No phenotype detected	Garrido-Martin et al. (2014)

Note that Cre-ER is only activated following tamoxifen treatment, which is given in adult life unless otherwise stated.

**Table 2:** **Summary of HHT phenotypes in conditional Acvlr1 knockout mouse models with different Cre lines**.

**Cre line**	**Targeted cell type (Cre expression)**	**HHT phenotype**	**Reference**
R26-Cre^ER^	All cell types	GI and lung hemorrhage. Dermal AVMs dependent on dermal wounding. 100% mortality	Park et al. (2009), Han et al. (2014)
L1-Cre	Arterial ECs of brain, lung, and GI tract (Cre active from late embryogenesis)	AVMs and hemorrhage in brain, lung, and GI tract. 100% mortality	Park et al. (2009)
Cdh5-Cre^ERT2^	ECs (Cre activated in neonate or adult).	*Neonatal*: retinal AVMs, lung hemorrhage, 100% mortality *Adult*: cecal hemorrhage, 100% mortality	Tual-Chalot et al. (2014)
Scl-Cre^ER^	ECs	Skin AVMs following wounding Visceral AVMs in absence of any additional external trigger 100% mortality	Garrido-Martin et al. (2014)
Myh11-Cre^ER^	vSMCs	No phenotype detected	Garrido-Martin et al. (2014)
Sm22α-Cre	vSMCs and ECs	Cerebral and spinal AVMs. High mortality	Milton et al. (2012)
NG2-Cre^ER^	Pericytes	No phenotype detected	Chen et al. (2014a)
Pdgfb-Cre^ER^	ECs	AVMs in brain following local VEGF stimulation; lung hemorrhage and visceral AVMs. 100% mortality	Chen et al. (2014a)

Note that Cre-ER is only activated following tamoxifen treatment, which is given in adult life unless otherwise stated.

**FIGURE 4:**
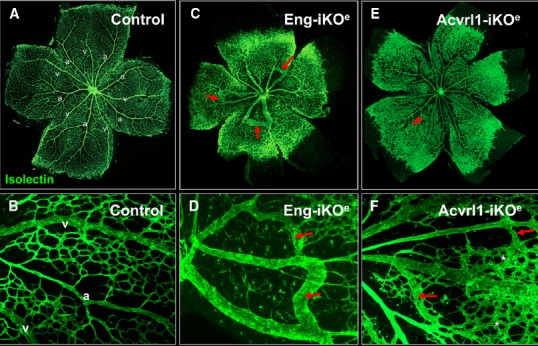
**Arteriovenous malformations in mouse neonatal retinas following endothelial specific loss of Eng or Acvrl1 expression.** Endothelial cells of the neonatal retinal are stained with isolectin to reveal normal two-dimensional architecture of the vascular plexus at postnatal day 7 (P7). Note the regular organization of arteries (a) and veins (v) with intervening capillary networks **(A)**, and at higher magnification **(B)**. Arteries are readily recognized by the capillary free zones around them **(A,B)**. Following endothelial specific depletion of endoglin (by tamoxifen treatment of Eng^fl/fl^; Cdh5-Cre^ERT2^ neonates), Eng-iKO^e^ retinal vessels at P7 show delayed progression of the vascular plexus toward the edge of the retina as well as abnormal connections between arteries and veins to generate multiple AVMs [red arrows in **(C)**, and shown in higher power images in **(D)**]. Endothelial specific loss of Acvrl1 leads to AVMs (red arrows in **E,F**), hyperbranching (**F**, asterisks) and enlargement of veins. Panels **(A,C)** are reproduced from a previous study where further details of the experimental approach can be found (Mahmoud et al., 2010; Tual-Chalot et al., 2014).

The effect of conditional knockdown of endoglin in ECs was first examined in detail using the mouse neonatal retina (Mahmoud et al., 2010). The mouse retina undergoes a well characterized process of blood vessel development during the first week of life. Retinal vessels arise from the center of the retina close to the optic nerve by postnatal day 1 (P1), and develop by angiogenesis (gradually growing outward from the optic disk toward the periphery) to form a highly organized vascular plexus that reaches the retinal periphery by P8 (Fruttiger, 2007). In this way, neonatal retinal blood vessels form a characteristic alternating pattern of arteries and veins with an intervening capillary network in a simple two-dimensional vascular plexus (Figure 4). This means that any abnormal angiogenesis events can be readily observed and characterized. In addition, using appropriate staining methods, individual ECs can be detected, permitting tracking of fundamental EC properties such as cell proliferation, arterial, and venous identity, cell-specific signaling as well as smooth muscle cell coverage. Endoglin knockdown in ECs results in a clear AVM phenotype in the neonatal retinal vasculature that is reminiscent of vascular malformations in HHT (Mahmoud et al., 2010). Furthermore, the retinal vasculature of these inducible endothelial specific endoglin knockout mice (*Eng*-iKO^e^) show delayed angiogenesis, an increased level of EC proliferation and enlarged veins (Figure 4). The arteriovenous connections have a venous phenotype and become more muscularized over time (Mahmoud et al., 2010), likely in response to increased blood flow. This is similar to the venous muscularization phenotype that was reported for dermal AVMs in HHT patients (Braverman et al., 1990). Moreover, delayed progression of the vascular plexus, is a very reproducible phenotype in the *Eng*-iKO^e^ retinas. Here, the delayed plexus is thicker than normal suggesting a defect in intercalation of ECs and a failure of the vessels to migrate and branch out correctly. No AVMs are observed when endoglin was depleted in ECs of adult mice, suggesting that developmental angiogenesis is an essential trigger for AVM formation, in line with the “three event” hypothesis (Figure 3).

A similar model using endothelial specific depletion of *Acvrl1* in early postnatal life (*Acvrl1*-iKO^e^) revealed a neonatal retinal vessel phenotype that overlaps with that of *Eng*-iKO^e^ neonates (Tual-Chalot et al., 2014). The overlapping features are venous enlargement, increased EC proliferation and AVMs (Figure 4). However, *Acvrl1*-iKO^e^ retinas show additional phenotypes that include vascular hyperbranching and loss of arterial identity. The vascular hyperbranching in the presence of reduced Acvrl1 signaling is suggested to be due to defects in the cross talk that normally occurs between Acvrl1 and Notch signaling pathways (Larrivee et al., 2012). Reduced Acvrl1 levels leads to reduced Smad1/5/8 signaling *in vivo* (Tual-Chalot et al., 2014) and this may also reduce Notch signaling, resulting in increased tip cell formation and hyperbranching. In addition, Eng expression (at both RNA and protein level) is markedly down-regulated in *Acvrl1*-depleted ECs indicating endoglin expression to be downstream of Acvrl1 signaling *in vivo* (Tual-Chalot et al., 2014) and in agreement with a previous study showing endothelial endoglin expression is downstream of BMP9 signaling (Scharpfenecker et al., 2007), likely through Acvrl1.

Conditional deletion of *Acvrl1* in vSMCs and a subpopulation of ECs using the SM22α-Cre transgene results in mice with AVMs in the brain and/or spinal cord. This is associated with paralysis or internal hemorrhages during the first 10–15 weeks of life, and a high degree of mortality, although some mice survive with multiple AVMs (Milton et al., 2012). As Cre is expressed in both vSMCs and ECs in this line, then it is not clear which is the responsible cell type. This was addressed in a more recent study that also compared the development of visceral and cutaneous AVMs in adult *Eng*- and *Acvrl1*-iKO mouse models (Garrido-Martin et al., 2014). Eng or Acvrl1 were first deleted from vascular ECs in adult life using Scl-CreER, then angiogenesis/inflammation was stimulated using a skin wounding model and this resulted (in both *Eng*- and *Acvrl1*-iKO^e^ models) in the formation of AVMs that were monitored in real time. In the *Acvrl1*-iKO^e^ mice the arteriovenous connections grew over time, but the dermal vessels of *Eng*-iKO^e^ mice exhibited a much more dynamic AVM remodeling (Garrido-Martin et al., 2014). The reason for these different vessel responses is not clear. It may be due to technical issues (e.g., different levels of *Eng* and *Acvrl1* knockout) or reflect slightly different roles of endoglin and Acvrl1 *in vivo*. Further work is required to discriminate between these possibilities. It is also important to emphasize here that Scl-CreER transgenic mice show minimal Cre recombinase activities in bone marrow derived cells in adult mice (Gothert et al., 2004; Garrido-Martin et al., 2014) suggesting that the AVMs are due to loss of Eng or Acvrl1 in ECs. However, it remains to be formally shown whether hematopoietic stem cells do significantly contribute to HHT disease. This is important because transfer of bone marrow from *Eng*^+/–^ mice was sufficient to cause cerebral vascular dysplasia following VEGF stimulation (Choi et al., 2013) and hematopoietic stem/progenitor cells appear to play a key role in vessel maturation (Goossens et al., 2011). The Cre lines used to date have only shown that loss of endoglin in LysM expressing macrophage is insufficient to cause cerebral AVMs following angiogenic stimulation (Choi et al., 2014; Table 1). Importantly, loss of endoglin or Acvrl1 in vSMCs had no detectable effect on the blood vessels (Garrido-Martin et al., 2014) consistent with work showing an EC requirement for these proteins during developmental angiogenesis (Mahmoud et al., 2010; Tual-Chalot et al., 2014). Independent studies also showed that endothelial depletion of endoglin or Acvrl1 was a prerequisite for brain AVM formation, whereas pericyte loss of Acvrl1 or macrophage specific loss of endoglin did not contribute to the AVM phenotype (Chen et al., 2014a; Choi et al., 2014). However, this does not rule out a potential contributory role of macrophage in AVM formation as monocytes and macrophage are associated with skin AVMs in HHT (Braverman et al., 1990) and may, for example, provide important pro-angiogenic signals. It is also of interest that one of these studies observed a mixed contribution of *Eng*-null and wild-type ECs in the induced brain vascular lesions (Choi et al., 2014), supporting the idea that HHT patient AVMs may be mosaic for affected and unaffected ECs.

Intriguingly, the GI tract appears to be an exception to the three event “rule” shown in Figure 3, because endothelial loss of endothelial *Acvrl1* alone is sufficient for GI bleeding (Tual-Chalot et al., 2014) and *Acvrl1*-iKO mice show AVMs in the absence of any overt pro-angiogenic trigger (Park et al., 2009). This could be a peculiar feature of the delicate GI vasculature which may have a high requirement for Acvrl1 function, or because there are subtle pro-angiogenic or inflammatory signals present (possibly triggered by the gut flora or tissue macrophage), which have not yet been formally evaluated in this model. In contrast, endothelial depletion of endoglin does not lead to AVMs in the GI vasculature (Garrido-Martin et al., 2014), and this phenotypic difference in the *Acvrl1*-iKO and *Eng*-iKO mouse models is also consistent with the increased frequency of bleeding GI lesions in HHT2 compared with HHT1 patients (Letteboer et al., 2006; Lesca et al., 2007; Komiyama et al., 2014).

## MOUSE MODELS OF HHT INDICATE THAT AVMs IN HHT PATIENTS WILL OCCUR FOLLOWING LOSS OF HETEROZYGOSITY

As discussed above, data from these more recent mouse models of HHT shows that homozygous loss of *Eng* or *Acvrl1* gene function is a prerequisite for AVM formation. This raises the question of how this might occur in HHT patients. One possibility is the (local) occurrence of somatic mutations in the remaining normal allele of *ENG* (for HHT1) or *ACVRL1* (for HHT2) to generate loss of heterozygosity (LOH). We propose that LOH due to a somatic second hit will inevitably precipitate susceptibility to disease pathology in HHT. This idea may also explain why clinical symptoms increase with age in HHT patients, as somatic mutations in *ENG* or *ACVRL1* would accrue. In fact, it was proposed some time ago that local LOH may be required to develop the vascular lesions seen in HHT (Berg et al., 1997). In this way, somatic mutations would increase the number of ECs that are null for endoglin (in HHT1) or null for *ACVRL1* (in HHT2) and following exposure to angiogenic or inflammatory signals these “null” ECs would be associated with vascular malformations (Figure 3). To our knowledge only one study has so far addressed this question in tissues from HHT1 patients (Bourdeau et al., 2000). The results show no local loss of endoglin protein expression in ECs within one cerebral AVM or one pulmonary AVM and it was therefore concluded that AVMs are unlikely to be due to loss of the second endoglin allele (Bourdeau et al., 2000). However, it is possible that those cells harboring the LOH mutation within the AV shunts could represent a small subpopulation of cells, in which case an immunohistochemical approach may not be sufficiently sensitive to detect a mosaic LOH. Interestingly, studies evaluating the pathogenesis of numerous related familial vascular malformations, including cerebral cavernous malformation, venous malformation, and glomuvenous malformation all reveal LOH mutations within a subset of cells comprising the vascular lesions (Brouillard and Vikkula, 2007; Akers et al., 2009; Limaye et al., 2009a,b). These findings reinforce the need for LOH to be re-assessed in vascular lesions from HHT patients using the advanced DNA sequencing techniques that are now available.

## IMAGING AVM FORMATION IN REAL TIME

**FIGURE 5:**
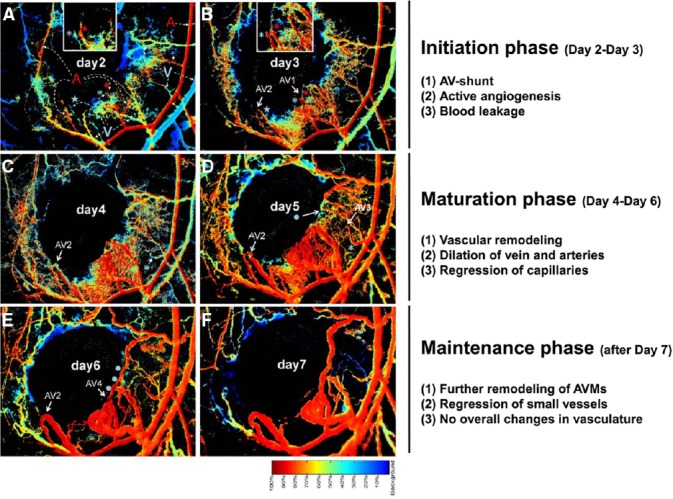
**Stages of AVM development in mouse skin AVMs following loss of Acvrl1 combined with wounding.** The novel connections between nascent arteries and veins can be seen by following hyperspectral imaging of the wound area in a dorsal window chamber in real time. The color bar indicates the relative oxygen saturation levels of the hemoglobin which allows tracking of arterial (red) and venous (blue) blood flow. Four novel AV shunts (AV1–4) appear over the course of 7 days following wounding. Arterial blood flowing into venous branches through AV shunts can be visualized. For instance, “blue” venous branches (marked by a blue asterisk or star) in day 2 **(A)** turned to “red” by connecting to adjacent arterial branches (marked by red asterisk for AV1 and star for AV2) in day 3 **(B)**, indicating establishment of AV shunts. A portion of this data and further methodological details can also be found in a previous publication (Han et al., 2014).

An important technological advance using spectral imaging of the dorsal murine skin allows AVM formation to be visualized in real time in a skinfold window chamber system (Park et al., 2009; Garrido-Martin et al., 2014; Han et al., 2014). The spectral imaging system detects the levels of hemoglobin saturation within any vessel segments to distinguish arterial from venous blood flow, and thus visual detection of AV shunting at the earliest stages is possible. The process of wound-induced AVM formation in the absence of *Acvrl1* can be divided into three phases; initiation, maturation, and maintenance (Han et al., 2014; Figure 5). In the initiation phase (2–4 days after wounding and *Acvrl1*-deletion), active angiogenic reaction is evident and AV shunts are established. In the maturation phase (4–6 days postwounding), blood vessels connected to the AV shunts begin to remodel, leading to dilatation of veins and arteries, and regression of capillaries and small vessels, probably due to hemodynamic changes. In the maintenance phase (7–9 days postwounding), further remodeling and regression of small vessels occurs but there are no overall changes in the structure of AV shunts. The process of wound-induced AVM formation in the absence of endothelial Eng appears to be more dynamic with arteriovenous shunts forming around day 6 after wounding and then regressing while new shunts formed nearby (Garrido-Martin et al., 2014). Combinations of brightfield and hyperspectral images and fluorescence video recording in this system is an important tool to examine the onset, progression, remodeling, and hemodynamic characters of these AV shunts in real time to reveal more about the development and progression of AVMs.

## MAIN CONCLUSIONS FROM THE MOUSE STUDIES

The main phenotypes of the conditional knockout mouse models examined to date (summarized in Tables 1 and 2) has led to the recognition that *the endothelial cell is the critical cell type in HHT*. Endothelial expression of Eng and Acvrl1 protects against AVM formation and hemorrhage, whereas loss of Eng or Acvrl1 in vSMCs, pericytes or macrophage does not lead to HHT symptoms. Furthermore, loss of Eng or Acvrl1 in ECs does not have to occur in all the ECs of an AVM and there may be mosaicism for loss of the target allele. Also, time is required for the evolution of an AVM in the mouse models: both dermal and retinal AVMs take a few days to form and subsequently enlarge over time.

The mouse models have also led to the proposal that three events (heterozygosity of *Eng* or *Acvrl1* mutation, plus LOH, plus a pro-angiogenic trigger) are necessary for AVM formation (Figure 3). We have called this the *three event hypothesis for AVM formation.* Loss of Eng or Acvrl1 in ECs is required but alone is not sufficient for AVM formation (with the exception of the GI tract AVMs in Acvrl1-iKO mice, discussed above). Heterozygosity of *ENG* or *ACVRL1* mutations represents the baseline situation in HHT1 and HHT2, respectively. Somatic mutations can then lead to total loss of either *ENG* or *ACVRL1* gene function in individual ECs or EC lineages. Alternatively, at least for ENG, there can be local transient loss of ENG protein due to protein shedding during inflammatory events. (We do not yet know whether Acvrl1 protein is also shed from ECs in inflamed tissues.) The third event required for AVM formation is a pro-angiogenic trigger. This may simply be the normal development process of forming new blood vessels during embryological or postnatal growth. Alternatively, angiogenesis may occur during pathological events, e.g., inflammatory angiogenesis, a possibility that aligns with the so called “response to injury paradigm” to explain why AVMs may follow pathologies or tissue trauma.

## STRENGTHS AND LIMITATIONS OF CURRENT MOUSE MODELS OF HHT

As discussed above and summarized in Tables 1 and 2, a range of different mouse models have been used to model the clinical features of HHT. The phenotypic outcomes largely depend on the Cre line being used and the stage of development or adult life when Cre is active to target *Eng* or *Acvrl1* genes. There are also technical issues such as how reproducibly the Cre-ER is expressed and activated; and how efficiently the target gene is “knocked out” in each case. For example the SM22α-Cre line has mosaic Cre activity in ECs during development, so AVMs occur in unpredictable locations, making it challenging to use these models for screening new therapies. In contrast, local wounding of the skin of *Acvrl1*-iKO mice in adult life leads to the formation of dermal AVMs in predictable sites and timescales that can be imaged in real time and used to test treatment strategies. For example, topical application of a VEGF-neutralizing antibody shortly after wounding can prevent the formation of AVMs in the skin of *Acvrl1*-iKO mice (Han et al., 2014). Alternatively, if VEGF is neutralized following initial AVM formation then it can block further progression of established AVMs or even cause regression of early AVMs (Han et al., 2014). Similarly, anti-VEGF treatment leads to attenuation of murine brain AVMs that form following the combination of local angiogenic stimulation and Acvrl1 depletion (Walker et al., 2012). These findings bode well for current anti-VEGF therapies in clinical trial for HHT patients (Kanellopoulou and Alexopoulou, 2013).

In terms of furthering our understanding of the events leading to AVM formation, the neonatal mouse retinal model provides a useful way to analyze the cellular and molecular changes that occur during development of a well characterized vascular plexus, while spectral imaging of adult mouse skin permits an overview of vascular remodeling in real time. Ultimately, technical advances in cell labeling and real time imaging will allow us to track individual ECs during formation of AVMs in these mouse models. In fact, the transparency of the zebrafish embryo already permits this type of study. Loss of Acvrl1 function leads to dilated cranial vessels that are characterized by increased EC numbers and stabilized by increased blood flow (Roman et al., 2002; Corti et al., 2011), suggesting that blood flow is a major factor in the maintenance and progression of AVMs. This remains to be formally shown in the mouse models of HHT.

A limitation of using mouse models is that it is not practical to investigate the pathogenicity of all individual HHT mutations, due to the cost and time required for detailed in vivo studies. However, the advent of new technologies to manipulate the mouse genome (e.g., CRISPR) means that these types of studies will become more and more efficient allowing the routine generation of animal models with patient-specific mutations. In the meantime, to meet this challenge, some patient specific cell based screens are available for diagnostic testing of patient specific mutations (Ricard et al., 2010; Mallet et al., 2014).

## HOW HAVE MOUSE MODELS OF HHT INFORMED CLINICAL CARE AND WHAT IS THEIR FUTURE POTENTIAL?

Evidence based medicine requires that clinical care is based on well informed decisions founded on robust research data from both clinical and basic science studies. The mouse models of HHT analyzed to date suggest that prevention of inflammation or angiogenesis would reduce the risk of AVM formation, while vascular stabilization agents would reduce the risk of hemorrhage. In addition, our increased understanding of disease mechanisms, including data from these mouse models, suggest that anti-inflammatory agents such as anti-TNFα therapy may be beneficial, at least for HHT1 patients, by reducing Eng protein shedding and protecting the vasculature during infections.

Furthermore, the most robust and reproducible mouse models of HHT are currently being used to develop therapies for prevention and for disease treatment. The *Acvrl*-iKO^e^ mouse provides a useful model for preclinical screening of potential therapies for GI bleeding, while *Eng*-iKO^e^ and *Acvrl1*-iKO^e^ mice provide valuable screening tools for evaluating therapies to reduce (or even reverse) AVM formation. Work showing the vascular stabilization role of thalidomide in a mouse model of HHT1 (Lebrin et al., 2010) underpins ongoing clinical trials to test whether thalidomide can reduce the severity of epistaxis in HHT. Furthermore, the extensive mouse model data revealing the critical role of angiogenesis triggers for AVM formation (Park et al., 2009; Mahmoud et al., 2010; Walker et al., 2011; Choi et al., 2012; Chen et al., 2014b; Garrido-Martin et al., 2014; Tual-Chalot et al., 2014) and the protective effect of anti-VEGF treatment (Han et al., 2014) supports promising findings from anti-angiogenic therapy using Bevacizumab in early clinical trials which appears to reduce the frequency and intensity of epistaxis, GI bleeding episodes, and liver AVMs, while topical administration reduces the frequency and the severity of epistaxis (Kanellopoulou and Alexopoulou, 2013).

## OUTSTANDING QUESTIONS AND FUTURE CHALLENGES

This review has focused on mouse models of HHT1 and HHT2. However, there are approximately 15–20% of HHT patients with no known *ENG* or *ACVRL1* mutations and ultimately new mouse models will be used to functionally investigate novel HHT genes once they have been identified. In the meantime, mouse models of HHT1 and HHT2 have reached an exciting stage and we now are in a strong position to examine many outstanding questions in the field; for example, whether AVMs always form in the same manner and at what point in their development they are reversible; whether EC proliferation always contributes to enlargement of arteriovenous shunts and to what extent the shunts are maintained, or exacerbated, by blood flow. Future work will reveal whether these mouse models can reproduce both the nidus-like “tangles” of vessels as well as the arteriovenous fistulae seen in HHT patients; and may tell us why AVMs are more common in lung and brain in HHT1 compared with HHT2 patients; or why the GI tract and liver are more susceptible to vascular malformations in HHT2. These and many more questions will be answered as the mouse models are further refined and investigations continue. We are confident that the next decade will bring important new insights into the understanding of HHT disease and we envisage a growing application of these models in pre-clinical screens of new therapies for HHT patients.

## AUTHOR CONTRIBUTIONS

All the authors substantially contributed to writing and editing the manuscript and have approved the final version.

### Conflict of Interest Statement

The authors declare that the research was conducted in the absence of any commercial or financial relationships that could be construed as a potential conflict of interest.
